# Anisotropic Collective Charge Excitations in Quasimetallic 2D Transition‐Metal Dichalcogenides

**DOI:** 10.1002/advs.201902726

**Published:** 2020-04-16

**Authors:** Chi Sin Tang, Xinmao Yin, Ming Yang, Di Wu, Jing Wu, Lai Mun Wong, Changjian Li, Shi Wun Tong, Yung‐Huang Chang, Fangping Ouyang, Yuan Ping Feng, Shi Jie Wang, Dongzhi Chi, Mark B. H. Breese, Wenjing Zhang, Andrivo Rusydi, Andrew T. S. Wee

**Affiliations:** ^1^ Department of Physics Faculty of Science National University of Singapore S12 Science Drive 3 Singapore 117551 Singapore; ^2^ NUS Graduate School for Integrative Sciences and Engineering National University of Singapore 21 Lower Kent Ridge Singapore 119077 Singapore; ^3^ Singapore Synchrotron Light Source (SSLS) National University of Singapore 5 Research Link Singapore 117603 Singapore; ^4^ Institute of Materials Research and Engineering (IMRE) A*STAR (Agency for Science, Technology and Research) 2 Fusionopolis Way, Innovis Singapore 138634 Singapore; ^5^ International Collaborative Laboratory of 2D Materials for Optoelectronics Science and Technology Shenzhen University Shenzhen 518060 China; ^6^ School of Physics and Electronics Central South University No. 932, South Lushan Road Changsha Hunan 410083 China; ^7^ Department of Materials Science & Engineering National University of Singapore 9 Engineering Drive 1 Singapore 117575 Singapore; ^8^ Bachelor Program in Interdisciplinary Studies National Yunlin University of Science and Technology 123 University Road, Section 3 Douliou Yunlin 64002 Taiwan

**Keywords:** anisotropic charge dynamics, phase transitions, plasmons, spectroscopic ellipsometry, transition‐metal dichalcogenides

## Abstract

The quasimetallic 1T′ phase 2D transition‐metal dichalcogenides (TMDs) consist of 1D zigzag metal chains stacked periodically along a single axis. This gives rise to its prominent physical properties which promises the onset of novel physical phenomena and applications. Here, the in‐plane electronic correlations are explored, and new mid‐infrared plasmon excitations in 1T′ phase monolayer WSe_2_ and MoS_2_ are observed using optical spectroscopies. Based on an extensive first‐principles study which analyzes the charge dynamics across multiple axes of the atomic‐layered systems, the collective charge excitations are found to disperse only along the direction perpendicular to the chains. Further analysis reveals that the interchain long‐range coupling is responsible for the coherent 1D charge dynamics and the spin–orbit coupling affects the plasmon frequency. Detailed investigation of these charge collective modes in 2D‐chained systems offers opportunities for novel device applications and has implications for the underlying mechanism that governs superconductivity in 2D TMD systems.

## Graphical abstract

New 1D mid‐infrared anisotropic plasmons are observed in the quasimetallic phase of WSe_2_ and MoS_2_ monolayers. In‐depth analysis reveals that the long‐range inter‐chain coupling is responsible for this 1D charge dynamics, and the plasmon energy is dictated by the effects of spin–orbit coupling. This study establishes an inextricable link between the 1D‐chain structure and charge dynamics in quasimetallic phase 2D transition‐metal dichalcogenides.

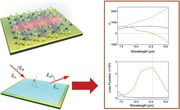

Low‐dimensional periodical patterned structures, such as 2D layered systems or 1D chain structures in higher dimensional materials, exhibit immensely intriguing wave phenomena due to the heavy influence by many‐body interactions.^[^ ^1^ ^]^ The highly correlated collective modes take the place of single‐particle excitations and detailed study of periodic materials kindles it as a burgeoning field of research spanning broad areas ranging from phononics, to photonics, plasmonics, and magnonics.^[^ ^2^, ^3^, ^4^ ^]^ A definitive example is the periodic CuO_2_ planes in copper oxide‐based (cuprate) systems where report is made that plasmon is induced via periodic interplanar interactions and this distinct collective mode is argued to play a pivotal role in mediating high‐temperature superconductivity.^[^ ^5^ ^]^ Another notable example is the report of the periodic infinite‐layer system Nd_0.8_Sr_0.2_NiO_2_, from which, by transforming the system from a perovskite structure to a 2D periodic layered structure, a new superconductivity state results in this nickelate system.^[^ ^6^ ^]^

With the continuous exploration of 2D transition‐metal dichalcogenides (TMDs), there arise novel high‐performance devices based on their remarkable electronic and optoelectronic properties.^[^ ^7^, ^8^, ^9^ ^]^ Besides the semiconducting 1H phase 2D TMDs, quasimetallic 1T′ phase 2D TMDs have particularly promised a range of new applications from supercapacitor electrodes^[^ ^9^ ^]^ to hydrogen‐based evolution reaction catalysts.^[^ ^7^ ^]^ The in‐plane atomic arrangement of 1T′ phase 2D TMDs comprises a distorted sandwich structure, where the transition‐metal atoms form a period‐doubling 2 × 1 structure comprising 1D zigzag chains.^[^ ^8^ ^]^ This novel 1D periodical structure gives rise to strong anisotropic properties that significantly influence the electronic properties of 2D TMDs. For instance, alongside the influences of spin–orbit coupling and electronic correlations, the anisotropic structure results in a band inversion around the Γ point near the Fermi level which leads to the fundamental and inverted gap opening.^[^ ^8^ ^]^ Distinct possibilities emerge in uncovering new physical phenomena in this unique structural phase. Specifically, unanswered questions remain on how 1D chain structures affect the charge dynamics of 1T′ phase 2D TMDs. With reports suggesting additional quasiparticle interactions can create strong‐correlated configurations yielding new phenomena such as Mott insulating system,^[^ ^10^ ^]^ superconductivity, and pseudogap phases in high‐temperature superconductors.^[^ ^11^ ^]^ Hence, it is vital to probe the quasiparticle dynamics in 1T′ phase 2D TMDs and unravel their correlated electronic properties.

Here, we report the direct observation of new mid‐infrared plasmons in 1T′ phase monolayer WSe_2_ and MoS_2_ which are absent from their semiconducting 1H phase counterparts. First‐principles investigation demonstrates that these plasmons are anisotropic while they are present in the direction perpendicular to the zigzag transition‐metal chain (*y*‐direction, **Figure** **1**a,b), they are absent along the zigzag chain. With the photon‐in‐photon‐out and photon energy specific methodology of high‐resolution spectroscopic ellipsometry, it is a premier technique to directly probe the plasmon modes (Figure 1c) where sample charging and higher harmonic processes can be ruled out. We deduce that coupling between the zigzag transition‐metal chains is the key mechanism driving the collective charge dynamics along the *y*‐direction (Figure 1b). Analysis indicates the significant role of the plasmons in the charge dynamics of 2D TMD quantum structures and mediating the onset of superconductivity reported in 1T′ phase 2D TMDs.^[^ ^12^ ^]^ This work also unveils the influence of spin–orbit coupling (SOC) in regulating the plasmon energy and establishes an link between the chain structure and 1D charge dynamics in 1T′ phase 2D TMDs.

**Figure 1:**
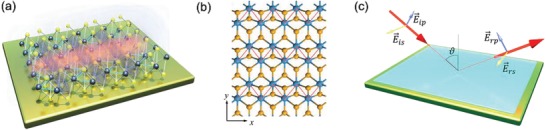
a) Mid‐infrared anisotropic plasmon in 1T′ phase WSe_2_. b) 1T′ phase monolayer WSe_2_ with its directional zigzag W structure traced by red dashed‐lines. c) Schematic of high‐resolution spectroscopic ellipsometry to probe the mid‐infrared optical properties of thin‐film systems.

Phase engineering techniques have made 1T′ phase 2D TMDs greatly accessible via various processes such as the *n*‐butyl lithium (*n*‐BuLi) treatment.^[^ ^13^ ^]^ However, large‐area fully covered 1T′ phase 2D TMDs are more favorable for the effective characterization of its ordered 1D chain structure via spectroscopic techniques. An annealing‐based technique for a high‐yield 1H‐1T′ phase transition of large‐area chemical vapour deposition‐grown 2D TMDs has recently been reported, where spectroscopic ellipsometry, Raman and photoluminescence spectroscopy are employed to confirm the different structural phases.^[^ ^13^ ^]^ Besides, this study has further indicated that that electron‐doping from the metallic substrate, facilitated by interfacial tensile strain, plays a very significant role in the induction of the 1H‐to‐1T′ phase transition of 2D TMDs.^[^ ^14^ ^]^ Prior study has reported that monolayer WSe_2_/Au annealed at the temperature region of ≈500–550 K enables a ≈59% yield of 1T'‐phase monolayer‐WSe_2_.^[^ ^15^ ^]^

High‐quality large‐area monolayer WSe_2_ samples are synthesized on sapphire substrate (detail in the Supporting Information). Each sample is annealed at the respective temperature in a high‐vacuum chamber with a base pressure of 1 × 10^−9^ mbar for ≈15 min. Thereafter, the sample is naturally cooled to room temperature before the measurement. Similar to previous reports,^[^ ^15^ ^]^ confirmation of the 1H‐1T′ phase transition of monolayer WSe_2_ on gold substrate is provided via a systematic annealing temperature‐dependent photoluminescence (PL), Raman, high‐resolution transmission electron microscopy (HRTEM) and UV–vis spectroscopic ellipsometry study. The characterization data of monolayer WSe_2_/Au using these three techniques are displayed in **Figure** **2**a–c (detail in the Supporting Information). The inverted and fundamental gaps are important features for 1T′ phase 2D TMDs.^[^ ^8^ ^]^ PL data shows the weakening exciton peaks (Figure 2a), the corresponding appearance of the characteristic 1T′ phase Raman modes (Figure 2b) and the 1T′ phase inverted gap feature (Figure 2c) take place mainly after annealing at 500 K (detail in the Supporting Information). It is important to highlight that the broad mid‐gap feature at ≈1600 nm from the UV–vis spectroscopic ellipsometry data (Figure 2c) has contributions from both the 1T′ phase inverted gap and the presence of charge–lattice interaction at the interface.^[^ ^14^ ^]^ While thermal decomposition begins after sample annealing at 550 K (Figure 2d), interfacial strain and electron doping by the Au substrate on the monolayer are still present. Therefore, there is still a small broad mid‐gap feature at ≈1600 nm after annealing at 550 K.

**Figure 2:**
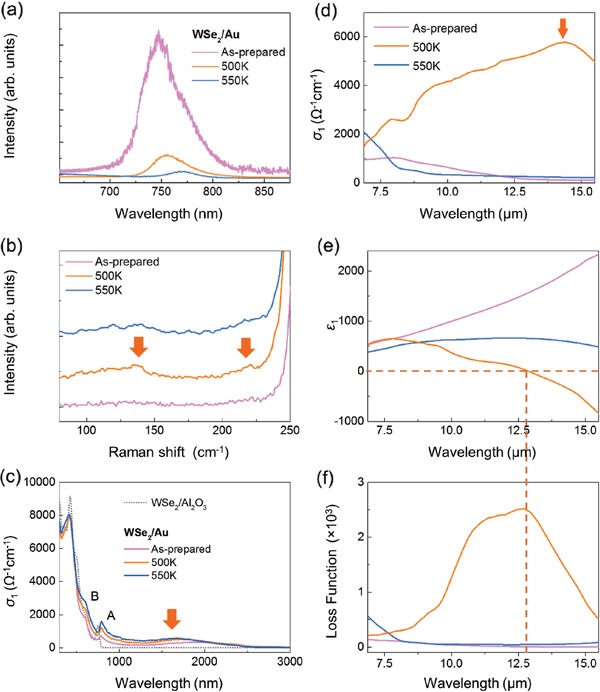
a) PL data of the monolayer WSe_2_/Au sample. b) Raman spectra where the orange arrows indicate the Raman features of 1T′ phase WSe_2_ after annealing at 500 K. c) IR‐to‐visible range spectroscopic ellipsometry data where the orange arrow indicates the position of the inverted gap feature of 1T′ phase monolayer WSe_2_ after annealing at 500 K. d) Optical conductivity, *σ*_1_, spectra of monolayer WSe_2_/Au sample with the orange arrow indicating the position of the fundamental gap of 1T′ phase monolayer WSe_2_ after annealing at 500 K. e) Dielectric function, *ε*_1_, and f) LF spectra of as‐prepared monolayer WSe_2_/Au and after annealing at respective temperatures. Intersection of the orange dashed lines matches the zero‐crossing position in e) the *ε*_1_ spectrum and f) the LF peak position.

Further confirmation of the 1H‐1T′ transition is made via HRTEM characterization that compares molecular configuration before and after the annealing process at 500 K (detail in the Supporting Information). Apart from confirming the 1H‐1T′ structural phase transition of the monolayer sample, these experiments further affirms the good monolayer crystalline property in its pristine state and after the annealing process. Figure 2d displays the optical conductivity, *σ*_1_, of monolayer WSe_2_/Au in its as‐prepared state and after annealing at 500 and 550 K, respectively. A strong peak feature with maximum intensity at ≈14.4 µm (≈0.09 eV) appears after annealing at 500 K but disappears after annealing at 550 K. This peak position is in agreement with the calculated fundamental gap size of ≈0.08 eV (≈15 µm) for 1T′ phase WSe_2_.^[^ ^8^ ^]^ While 1T′ phase monolayer WSe_2_ is present after annealing at 500 K, the start of thermal decomposition brought about by high heat leads to the disappearance of the fundamental gap after annealing at 550 K. This is an indication that the fundamental gap feature is easily affected by decomposition and defects due to its proximity to the Fermi Level. The 1H‐1T′ phase transition is confirmed via the direct observation of the inverted and fundamental gaps. Furthermore, this annealing temperature is consistent with previous studies where ≈500 K is near the optimum temperature for the 1H‐1T′ phase transition of WSe_2_/Au.^[14,15]^

Having confirmed the 1T′ phase monolayer WSe_2_ after sample annealing at 500 K, we analyze the *ε*_1_ spectra (Figure 2e) simultaneously with its loss function (LF) spectra (Figure 2f). Unlike the as‐prepared state, the LF spectrum registers a distinct main peak at ≈12.7 µm that is absent from the *σ*_1_ and *ε*_1_ spectra (Figure 2d,e), which also coincides with the zero‐crossing of the corresponding *ε*_1_ spectrum at ≈12.8 µm (Figure 2f). To understand these optical features in the LF spectra, we consider the phenomenon of plasmon excitation–quantum of collective charge excitation arising from interactions between electromagnetic fields and charges.^[^ ^16^ ^]^ Such intense collective charge excitation manifests itself as a prominent peak in the LF spectrum at a characteristic plasma frequency, *ω*_p_, which depends on the carrier density and the media's complex dielectric response. The presence of a zero‐crossing in the real dielectric function component, *ε*_1_(*ω*_p_) = 0, confirms the presence of the intense plasmon mode.^[^ ^16^ ^]^ The collective optical features in both LF and *ε*_1_ spectra of 1T′ phase WSe_2_/Au strongly suggest the appearance of a plasmon mode in the mid‐infrared regime at ≈12.7 µm. Note that the slight disparity between the significant LF peak and the *ε*_1_ zero‐crossing positions is attributed to charge scattering present with the 2D lattice.

Plasmon excitations are generally reported in metals,^[^ ^17^ ^]^ including graphene^[^ ^18^ ^]^ where Drude responses are observed. For metals, the plasma frequency is usually located in the ultraviolet regime,^[^ ^17^ ^]^ while electromagnetic waves with frequency above the plasma frequency is transmitted because the electrons in the material are unable to respond swiftly enough to screen, light frequencies below the plasma frequency are reflected due to the electrodynamical interaction with the material where the electric field is screened by the electrons. Interestingly, this monolayer WSe_2_ system comprises both 1H phase (with bandgap of ≈1.6 eV) and 1T′ phase structure (with fundamental gap of ≈0.09 eV), does not have a Drude response (Figure 2d), indicating that our sample is not truly metallic but possesses a mid‐infrared plasmon. Hence, the experimentally observed plasmon excitation at ≈12.7 µm may be associated with the 1T′ phase anisotropic structure. A comprehensive first‐principles study is conducted to substantiate the connection between the anisotropic 1T′ phase WSe_2_ structure and the new mid‐infrared plasmon.

Fitting analyses show that the plasmon peak widths (detail in Table S1, Supporting Information) are similar to those reported in noble metals^[^ ^17^ ^]^ and graphene nanostructures.^[^ ^19^ ^]^ Interestingly, the dephasing time, *T*, of these plasmons are significantly higher than those of other systems (detail in the Supporting Information).^[^ ^19^, ^20^ ^]^ This suggests the 1T′ phase 2D TMD system is less prone to plasmonic dissipation and other charge scattering processes due to interactions with lattice and site defects. Hence, such monolayer systems hold potential for low‐loss novel device applications. Besides, such analyses to charge scattering in 2D TMDs are pivotal to the understanding of possible mitigating factors to reduce plasmonic dissipation and losses.

To further confirm this observation, first‐principles calculations are performed. **Figure** **3**a,b display the calculated *ε*_1_ and LF spectra of 1T′ phase monolayer WSe_2_ with SOC effects accounted for (detail in the Supporting Information). While Figure 3a displays a zero‐crossing at ≈4.13 µm, importantly, a prominent peak is noticeable in the calculated LF spectrum (Figure 3b) which resolves the 1T′ phase monolayer WSe_2_ plasmon along the zigzag transition‐metal chain direction (*x*‐direction) and the direction perpendicular to it (*y*‐direction) depicted in Figure 1b. Interestingly, while no feature is observed along the *x*‐direction, this prominent peak is present along the *y*‐axis at ≈4.6 µm. This is a substantial theoretical proof of the new mid‐infrared plasmon in 1T′ phase monolayer WSe_2._ With no other peaks present at longer wavelength (lower energy close to Fermi level, Figure 3b inset), it suggests further agreement with the experimentally observed mid‐infrared plasmon in 1T′ phase monolayer WSe_2_. Besides, the result provides strong evidence that this plasmon possesses anisotropic features. It only occurs in the direction perpendicular to the zigzag transition‐metal chain.

**Figure 3:**
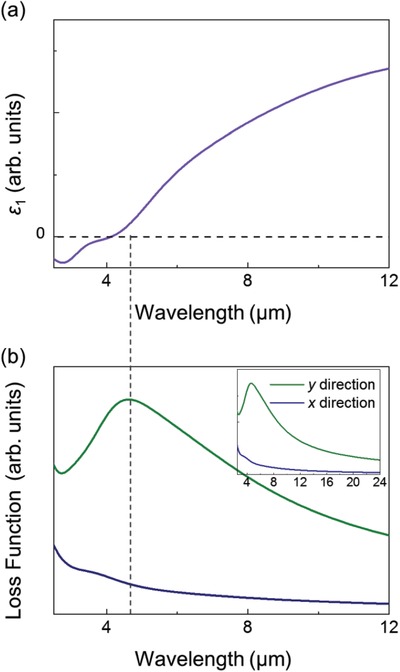
a) *ε*_1_, and b) axis‐dependent LF spectra derived via first‐principles calculations. (Inset: Calculated LF spectra extended to 18 µm.) Dashed‐lines are visual guides to locate plasmon peak position with respect to the *ε*_1_ spectrum zero‐crossing.

Discrepancy of the plasmon position between the experimental and first‐principles study is attributed to the sample's intrinsic defects and interfacial localized strain which distorts the regular zigzag 1T′ chains due to the nonuniform interactions with the metallic substrate and SOC effects.^[^ ^14^, ^21^ ^]^ The blue shift in the plasmon peak position of monolayer WSe_2_ without SOC effects (Figure S3, Supporting Information) shows that the disparity in plasmon position with experimental result further increases by blue shifting to ≈3.6 µm. This demonstrates how SOC affects the plasmon energy of 2D TMDs. Besides, the optoelectronic features of the monolayer WSe_2_ are tunable by intrinsic film defects and effects of localized interfacial lattice strain while the first‐principles study models a perfect 2D lattice.

Recently, the interplanar Coulomb interaction and 2D charge dynamics are identified as causes for the coherent acoustic plasmon in cuprate superconductors.^[^ ^5^ ^]^ This long‐range coupling between the CuO_2_ planes and the propagation of the plasmons is suggested to play a crucial role in mediating superconductivity. With the interlayer coupling between 2D CuO_2_ planes in 3D cuprate lattices, such analogous phenomenon involving reduced dimensionality is also noticeable here where coupling between 1D zigzag chains in 2D TMDs occurs. Hence, the coupling between zigzag transition‐metal chains in 1T′ phase monolayer WSe_2_ drives the collective 1D charge dynamics (Figure 1a) which eventually results in the plasmon. The notion that long‐range interchain coupling leads to plasmon formation is further substantiated by reports that long‐range electronic correlations leads to the appearance of plasmons in other strongly correlated systems.^[^ ^22^ ^]^

Having confirmed the mid‐infrared plasmon in 1T′ phase monolayer WSe_2_, we further demonstrate that it also appears in other 1T′ phase 2D TMDs where similar experimental process is conducted on 1T′ phase monolayer‐MoS_2_. 1H‐1T′ phase transition for monolayer‐MoS_2_/Au is confirmed via PL, Raman spectroscopy, and spectroscopic ellipsometry displayed in **Figure** **4**a–c (detail in the Supporting Information). Spectroscopic ellipsometry results show the appearance of the inverted gap (1T′ phase) as a mid‐gap peak (Figure 4c). Figure 4d–f displays the optical conductivity, dielectric function, and LF, of monolayer‐MoS_2_/Au in its as‐prepared state and after annealing at 500 and 550 K characterized using infrared‐range Spectroscopic ellipsometry.

**Figure 4:**
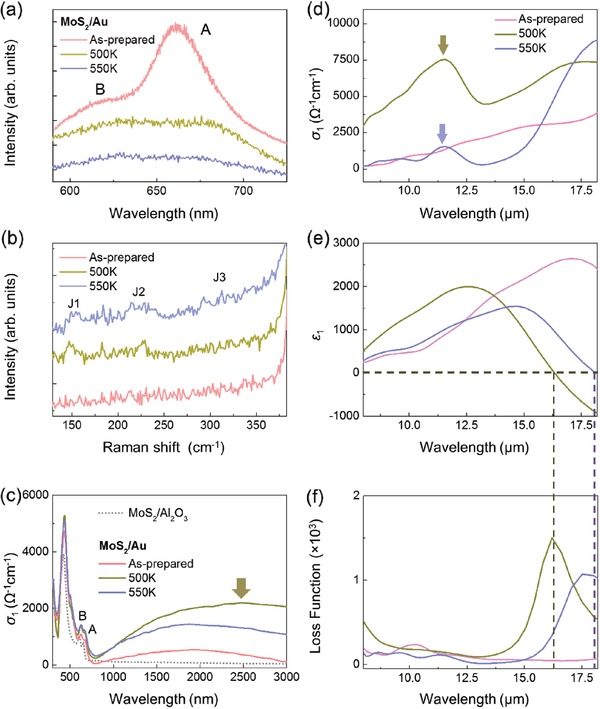
a) PL data of the monolayer‐MoS_2_/Au sample. b) Raman spectra where the J1, J2, and J3 features of 1T′ phase monolayer‐MoS_2_ are indicated. c) IR‐to‐visible range spectroscopic ellipsometry data where the arrow indicates the position of the inverted gap feature of 1T′ phase monolayer‐MoS_2_/Au. d) Optical conductivity, *σ*_1_, spectra of monolayer‐MoS_2_/Au where color‐coded arrows indicate the position of the fundamental gap of 1T′ phase MoS_2_/Au after annealing at the respective temperature. e) Dielectric function, *ε*_1_, and f) LF spectra of as‐prepared monolayer‐MoS_2_/Au and after annealing at respective temperatures. Intersections of the dashed lines match the zero‐crossing positions of e) the *ε*_1_ spectra and f) the LF peak positions after annealing at the respective temperature.

In its as‐prepared state, the *σ*_1_ spectrum of monolayer‐MoS_2_/Au is consistent with a previous study.^[^ ^14^ ^]^ By annealing the sample at 500 K, a peak appears at ≈11.5 µm (color‐coded arrows in Figure 4d). While the peak intensity reduces after annealing at 550 K, it persists at the same wavelength. Similar to previous annealing‐based study, this peak is attributed to the fundamental gap that is formed due to the phase transition.^[^ ^14^ ^]^ The fundamental gap position at ≈11.5 µm (≈0.1 eV) is consistent with the previous theoretical study of monolayer‐MoS_2_.^[^ ^8^ ^]^ Observations of both the inverted and fundamental gaps confirmed the phase transition.

Having ascertained the presence of the 1T′ phase monolayer‐MoS_2_ after annealing, analysis of the *ε*_1_ and LF spectra (Figure 4e,f, respectively) was performed to demonstrate the presence of the plasmon mode. After annealing at 500 K, the *ε*_1_ spectrum zero‐crossing is observed at ≈16.3 µm (Figure 4e) alongside the appearance of a main broad LF peak at ≈16.2 µm which is absent from both the *ε*_1_ and *σ*_1_ spectra. After annealing at 550 K, the main LF peak is red shifted to ≈17.7 µm while the *ε*_1_ zero‐crossing at ≈18.1 µm. Hence, the mid‐infrared plasmon is also present in 1T′ phase monolayer‐MoS_2_. Table S1 in the Supporting Information summarizes the plasmon fitting properties for both 1T′ phase monolayer WSe_2_ and MoS_2_. Note the plasmon energy of WSe_2_ (≈12–13 µm) is higher than that of MoS_2_ (≈16–18 µm), with the SOC strength directly related to the atomic mass of the constituent atoms,^[^ ^23^ ^]^ SOC effects is stronger in monolayer WSe_2_ than monolayer‐MoS_2_. Hence, SOC effects may play a crucial role in plasmon formation and in determining its position.

This study holds important implications in unravelling the mechanism governing superconductivity in 1T′ phase 2D TMDs.^[^ ^12^, ^24^ ^]^ Multiple models are proposed to identify the mechanism providing the “pairing glue” for the formation of electron pairs that mediates superconductivity. For instance, the role of phonons in charge–lattice interactions^[^ ^25^ ^]^ or magnons in spin fluctuations^[^ ^11^ ^]^ have been considered as the “pairing glue.” With suggestions of interplanar plasmons in multilayer cuprates mediating high‐temperature superconductivity,^[^ ^26^ ^]^ we postulate that plasmons in 1T′ phase 2D TMDs could facilitate the electron–plasmon interaction mechanism to form electron pairs that possibly underlies their superconductive properties^[^ ^12^, ^24^ ^]^ (detail in the Supporting Information). This is further substantiated by reports made that 1D chains have been associated with superconductivity.^[^ ^27^, ^28^ ^]^

Overall, we provide an exceptional opportunity to study the 1D behavior of mid‐infrared plasmons in monolayer WSe_2_ and MoS_2_ unique to their 1T′ phase. While 1H‐1T′ phase transition is achieved via our annealing technique, the reverse process is also readily accessible.^[^ ^13^, ^29^ ^]^ Therefore, the ease of regulating the 1H‐1T′ phases in 2D TMDs allows one to tune this system as a mid‐infrared plasmon on/off‐switch detector. Thus, it serves as an effective plasmonic photodetector which promises novel nanoplasmonic applications and other heterostructure engineering devices in the optical and near‐infrared regime. This new mid‐infrared plasmon open a new way in the fabrication of optoelectronic devices where plasmons can be exploited in multiple scientific and engineering applications, as compared to the plasmons in normal metals which are usually found in the ultraviolet range. Further analysis suggests that collective charge dynamics is induced by the long‐range coupling between the zigzag transition‐metal chains in 1T′ phase 2D TMDs. This unravels the role of long‐range electronic correlations in 2D TMD systems. In‐depth study of the significantly higher plasmon dephasing times also provide clues to possible mitigating factors for reducing plasmonic dissipation and losses in 2D TMD systems.

## Conflict of Interest

The authors declare no conflict of interest.

## Author Contributions

C.S.T., X.Y., and M.Y. contributed equally to this work. C.S.T., X.Y., L.M.W., and S.J.W. performed spectroscopic ellipsometry measurements; D.W. F.O., and W.Z. prepared high‐quality monolayer‐films and performed Raman and Photoluminescence spectroscopic measurements; M.Y. and Y.P.F. carried out the first principles calculation; C. L. performed HRTEM and relevant data analysis on structural transition. C.S.T., X.Y., and M.Y. analyzed the data and wrote the manuscript with assistance from all authors. X.Y. and A.T.S.W. conceived and supervised the project.

## Supporting information

Supporting Information

## References

[1] D. Boies , C. Bourbonnais , A. M. S. Tremblay , Phys. Rev. Lett. 1995, 74, 968. 10058894 10.1103/PhysRevLett.74.968

[2] M. Maldovan , Nature 2013, 503, 209. 24226887 10.1038/nature12608

[3] A. V. Chumak , V. I. Vasyuchka , A. A. Serga , B. Hillebrands , Nat. Phys. 2015, 11, 453.

[4] P. Lodahl , S. Mahmoodian , S. Stobbe , A. Rauschenbeutel , P. Schneeweiss , J. Volz , H. Pichler , P. Zoller , Nature 2017, 541, 473. 28128249 10.1038/nature21037

[5] M. Hepting , L. Chaix , E. W. Huang , R. Fumagalli , Y. Y. Peng , B. Moritz , K. Kummer , N. B. Brookes , W. C. Lee , M. Hashimoto , T. Sarkar , J. F. He , C. R. Rotundu , Y. S. Lee , R. L. Greene , L. Braicovich , G. Ghiringhelli , Z. X. Shen , T. P. Devereaux , W. S. Lee , Nature 2018, 563, 374. 30429543 10.1038/s41586-018-0648-3

[6] D. Li , K. Lee , B. Y. Wang , M. Osada , S. Crossley , H. R. Lee , Y. Cui , Y. Hikita , H. Y. Hwang , Nature 2019, 572, 624. 31462797 10.1038/s41586-019-1496-5

[7] T. F. Jaramillo , K. P. Jørgensen , J. Bonde , J. H. Nielsen , S. Horch , I. Chorkendorff , Science 2007, 317, 100. 17615351 10.1126/science.1141483

[8] X. Qian , J. Liu , L. Fu , J. Li , Science 2014, 346, 1344. 25504715 10.1126/science.1256815

[9] M. Acerce , D. Voiry , M. Chhowalla , Nat. Nanotechnol. 2015, 10, 313. 25799518 10.1038/nnano.2015.40

[10] M. Endres , M. Cheneau , T. Fukuhara , C. Weitenberg , P. Schauß , C. Gross , L. Mazza , M. C. Bañuls , L. Pollet , I. Bloch , S. Kuhr , Science 2011, 334, 200. 21998381 10.1126/science.1209284

[11] D. J. Scalapino , Rev. Mod. Phys. 2012, 84, 1383.

[12] Y. Qi , P. G. Naumov , M. N. Ali , C. R. Rajamathi , W. Schnelle , O. Barkalov , M. Hanfland , S.‐C. Wu , C. Shekhar , Y. Sun , V. Süß , M. Schmidt , U. Schwarz , E. Pippel , P. Werner , R. Hillebrand , T. Förster , E. Kampert , S. Parkin , R. J. Cava , C. Felser , B. Yan , S. A. Medvedev , Nat. Commun. 2016, 7, 11038. 26972450 10.1038/ncomms11038 PMC4793082

[13] G. Eda , H. Yamaguchi , D. Voiry , T. Fujita , M. Chen , M. Chhowalla , Nano Lett. 2011, 11, 5111. 22035145 10.1021/nl201874w

[14] X. Yin , Q. Wang , L. Cao , C. S. Tang , X. Luo , Y. Zheng , L. M. Wong , S. J. Wang , S. Y. Quek , W. Zhang , A. Rusydi , A. T. S. Wee , Nat. Commun. 2017, 8, 486. 28883392 10.1038/s41467-017-00640-2 PMC5589873

[15] X. Yin , C. S. Tang , D. Wu , W. Kong , C. Li , Q. Wang , L. Cao , M. Yang , Y.‐H. Chang , D. Qi , F. Ouyang , S. J. Pennycook , Y. P. Feng , M. B. H. Breese , S. J. Wang , W. Zhang , A. Rusydi , A. T. S. Wee , Adv. Sci. 2019, 6, 1802093. 10.1002/advs.201802093 PMC6446595 30989029

[16] D. Pines , Rev. Mod. Phys. 1956, 28, 184.

[17] M. Rocca , F. Moresco , U. Valbusa , Phys. Rev. B 1992, 45, 1399. 10.1103/physrevb.45.1399 10001618

[18] A. N. Grigorenko , M. Polini , K. S. Novoselov , Nat. Photonics 2012, 6, 749.

[19] H. Yan , T. Low , W. Zhu , Y. Wu , M. Freitag , X. Li , F. Guinea , P. Avouris , F. Xia , Nat. Photonics 2013, 7, 394.

[20] J. van Wezel , R. Schuster , A. König , M. Knupfer , J. van den Brink , H. Berger , B. Büchner , Phys. Rev. Lett. 2011, 107, 176404. 22107547 10.1103/PhysRevLett.107.176404

[21] A. Allain , A. Kis , ACS Nano 2014, 8, 7180. 24949529 10.1021/nn5021538

[22] E. G. C. P. van Loon , H. Hafermann , A. I. Lichtenstein , A. N. Rubtsov , M. I. Katsnelson , Phys. Rev. Lett. 2014, 113, 246407. 25541788 10.1103/PhysRevLett.113.246407

[23] F. Herman , C. D. Kuglin , K. F. Cuff , R. L. Kortum , Phys. Rev. Lett. 1963, 11, 541.

[24] C. Guo , J. Pan , H. Li , T. Lin , P. Liu , C. Song , D. Wang , G. Mu , X. Lai , H. Zhang , W. Zhou , M. Chen , F. Huang , J. Mater. Chem. C 2017, 5, 10855.

[25] J. Bardeen , L. N. Cooper , J. R. Schrieffer , Phys. Rev. 1957, 108, 1175.

[26] V. Z. Kresin , H. Morawitz , Phys. Rev. B 1988, 37, 7854. 10.1103/physrevb.37.7854 9944091

[27] E. Berg , T. H. Geballe , S. A. Kivelson , Phys. Rev. B 2007, 76, 214505.

[28] A. P. Petrović , D. Ansermet , D. Chernyshov , M. Hoesch , D. Salloum , P. Gougeon , M. Potel , L. Boeri , C. Panagopoulos , Nat. Commun. 2016, 7, 12262. 27448209 10.1038/ncomms12262 PMC4961838

[29] S. S. Chou , Y.‐K. Huang , J. Kim , B. Kaehr , B. M. Foley , P. Lu , C. Dykstra , P. E. Hopkins , C. J. Brinker , J. Huang , V. P. Dravid , J. Am. Chem. Soc. 2015, 137, 1742. 25608577 10.1021/ja5107145

