# Supplementary material for: Anisotropic Collective Charge Excitations in Quasimetallic 2D Transition‐Metal Dichalcogenides
Source: Adv Sci (Weinh). 2020 Apr 16;7(10):1902726. doi: 10.1002/advs.201902726 (PMC7237846; doi:10.1002/advs.201902726)
Supplement: Supplementary file 1 — Supporting Information [file ADVS-7-1902726-s001.pdf]

WILEY-VCH

Copyright WILEY-VCH Verlag GmbH & Co. KGaA, 69469 Weinheim, Germany, 2020.

Supporting Information

Anisotropic collective charge excitations in quasi-metallic two-dimensional transition-metal dichalcogenides

Chi Sin Tang[†]*, Xinmao Yin*[†,]**, Ming Yang*[†]*, Di Wu, Jing Wu, Lai Mun Wong, Changjian Li, Shi Wun Tong, Yung-Huang Chang, Fangping Ouyang, Yuan Ping Feng, Shi Jie Wang, Dongzhi Chi, Mark B. H. Breese, Wenjing Zhang, Andrivo Rusydi, Andrew T. S. Wee**

*Correspondence to phyyxm@nus.edu.sg (Y. X.), phyweets@nus.edu.sg (A.T.S.W.).

†These authors contributed equally to this work.

Supplementary Figures

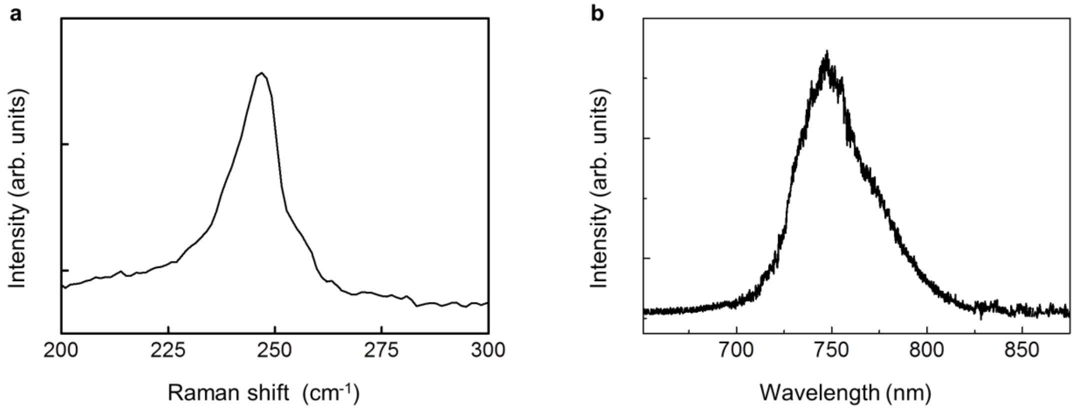

Figure S1. Raman and Photoluminescence spectra of the CVD-grown WSe_2 monolayer. (a) Raman spectra, where the two characteristic peaks for monolayer-WSe_2 at ~249cm^{-1} (E^1_{2g}-mode), and ~259cm^{-1} (A_{1g}-mode). (b) Photoluminescence spectra, a strong emission at ~752 nm corresponding to exciton A of monolayer-WSe_2.

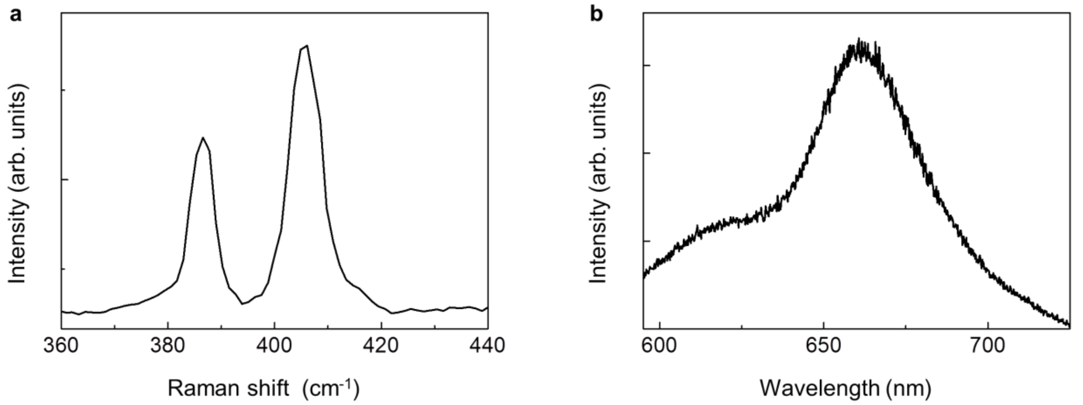

Figure S2. Raman and Photoluminescence spectra of CVD-grown monolayer-MoS_2 on sapphire. (a) Raman spectra, where the two characteristic peaks for monolayer-MoS_2 at ~392 cm^{-1} (E^1_{2g}-mode), and ~413cm^{-1} (A_{1g}-mode). (b) Photoluminescence spectra, strong emissions at ~670nm and ~618nm corresponding to excitons A and B, respectively of monolayer-MoS_2.

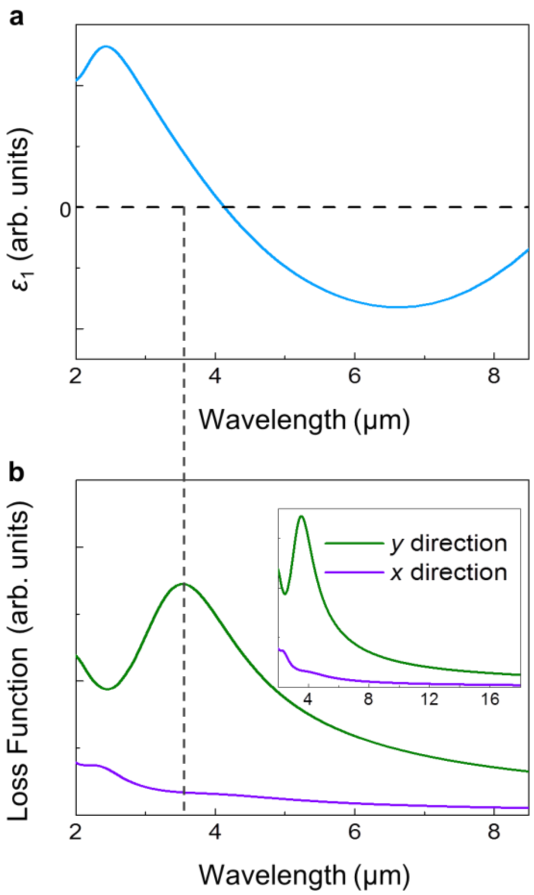

Figure S3. Optical features of 1T'-phase monolayer-WSe_2 without the influence of spin-orbit interaction derived via first-principles calculations. (a) ε_1 with zero-crossing at ~4.1 μm, and (b) axis-dependent Loss-function spectra with plasmon peak along the y-direction at ~3.5μm.

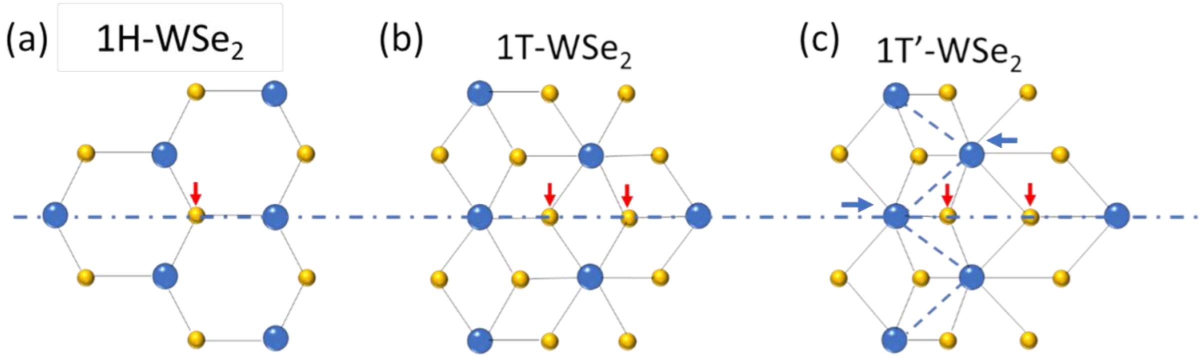

Figure S4. Top view of the molecular structure of monolayer-WSe_2. (a) 1H-, (b) 1T-,

and (c) 1T’-phase. Red vertical arrows indicate the positions of the Se-atoms while blue horizontal arrows in (c) indicate the distortion of the W-atoms in 1T’-phase WSe_2.

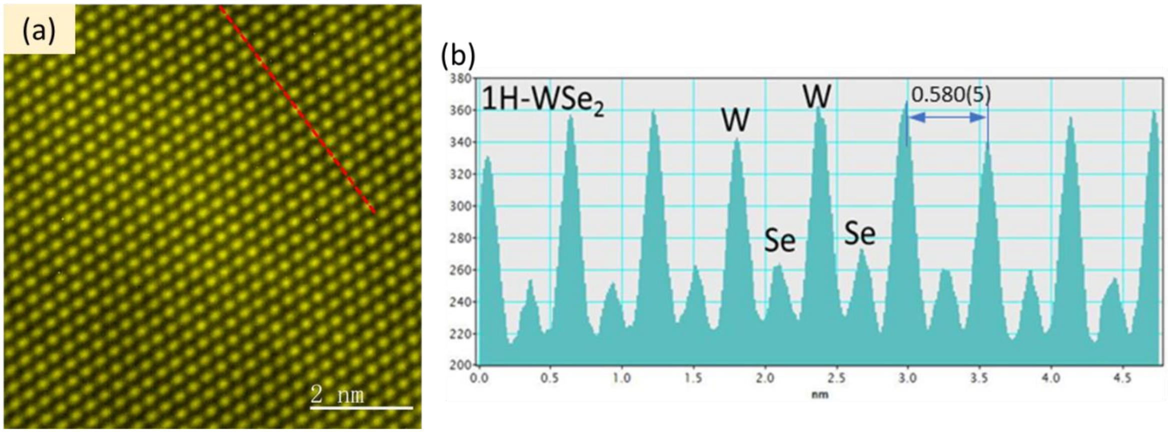

Figure S5. (a) HRTEM image of 1H-phase monolayer-WSe_2/Au before annealing. (b) Intensity profile along the red dashed line as indicated in (a) with the approximate length between adjacent W-atoms.

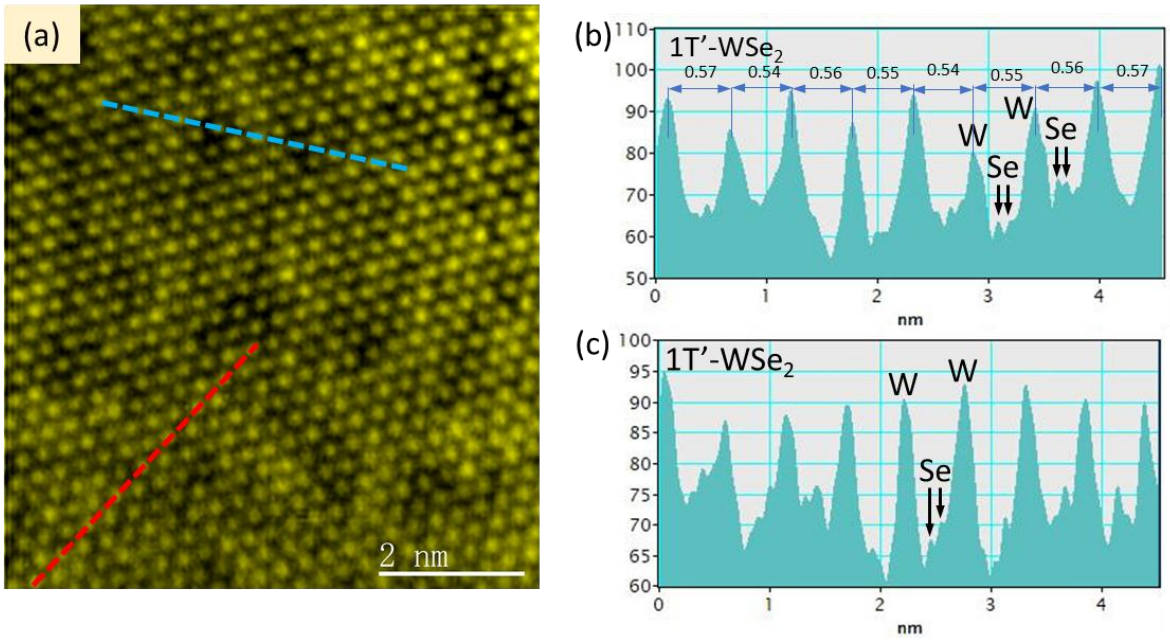

Figure S6. (a) HRTEM image of 1T’-phase monolayer-WSe_2/Au after annealing at 500K. (b) Intensity profiles along the blue dashed line as indicated in (a) with the approximate

length between adjacent W-atoms. (c) Intensity profiles along the red dashed line as indicated in (a).

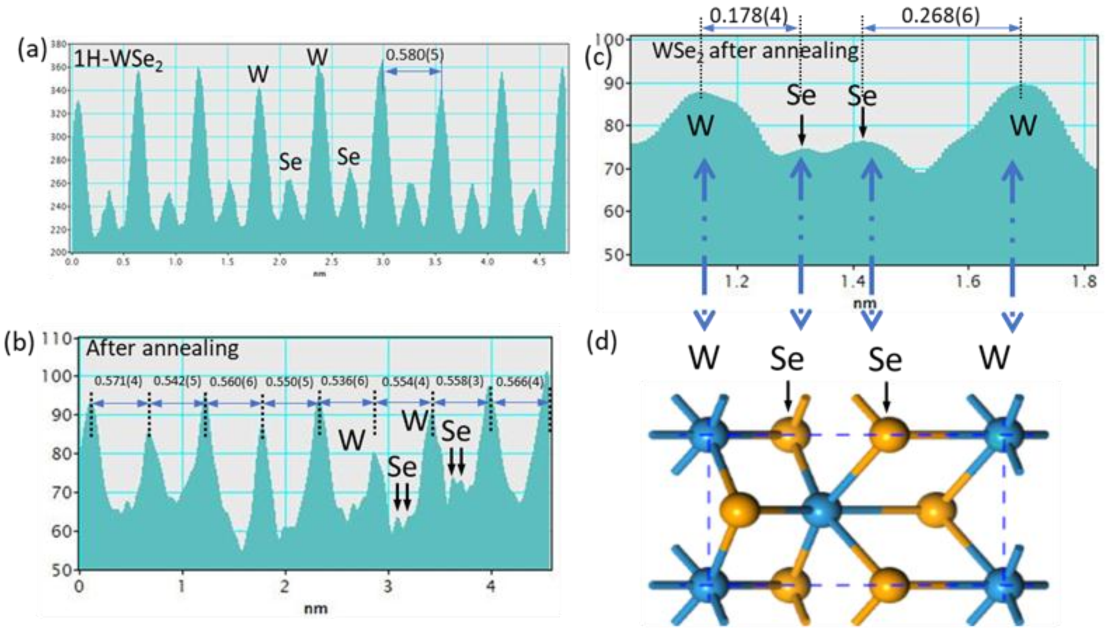

Figure S7. HR-TEM Intensity profiles of monolayer-WSe_2 in (a) 1H-, and (b) 1T'-phase. (c) Zoomed in HR-TEM intensity profiles of 1T'-phase WSe_2, and (d) top view of the molecular structure.

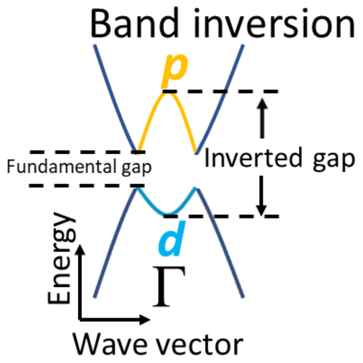

Figure S8. Schematic band structure of 1T'-phase 2D-TMDs. Lattice distortion along and strong electron-electron correlations in results in band inversion which in turn results in the opening of the fundamental and inverted gap.

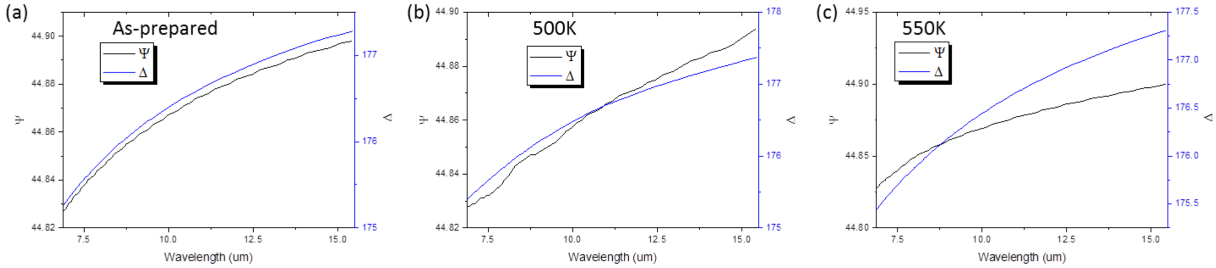

Figure S9. Ψ and Δ data of WSe_2/Au in the mid-IR regime. (a) As-prepared, (b) after annealing at 500K and, (c) after annealing at 550K.

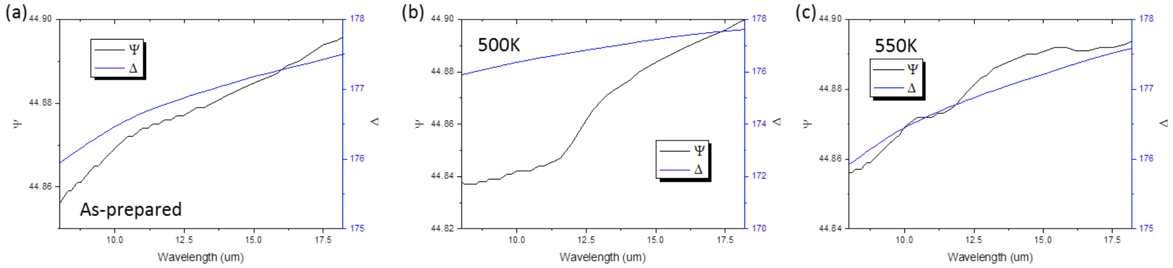

Figure S10. Ψ and Δ data of MoS_2/Au in the mid-IR regime. (a) As-prepared, (b) after annealing at 500K and, (c) after annealing at 550K.

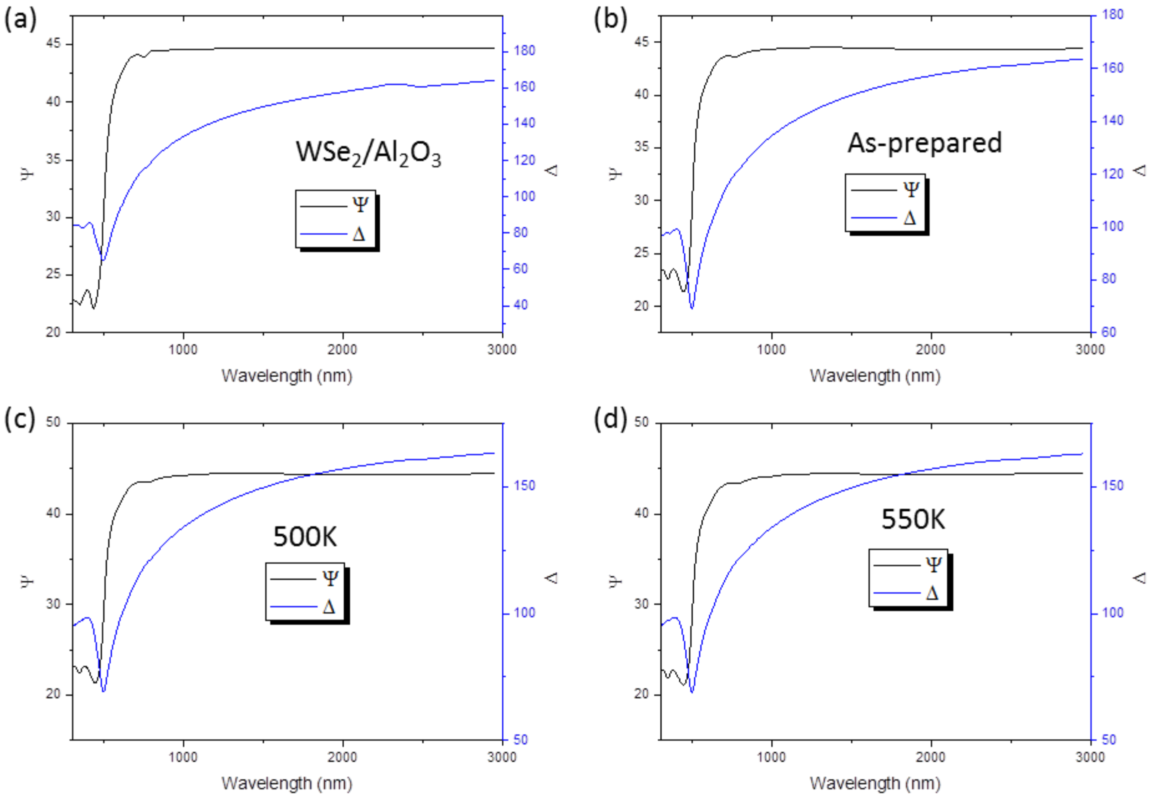

Figure S11. Ψ and Δ data of monolayer-WSe_2 in the near-IR-to-visible range. (a) WSe_2/Al_2O_3, (b) As-prepared WSe_2/Au, (c) WSe_2/Au after annealing at 500K and, (d) WSe_2/Au after annealing at 550K.

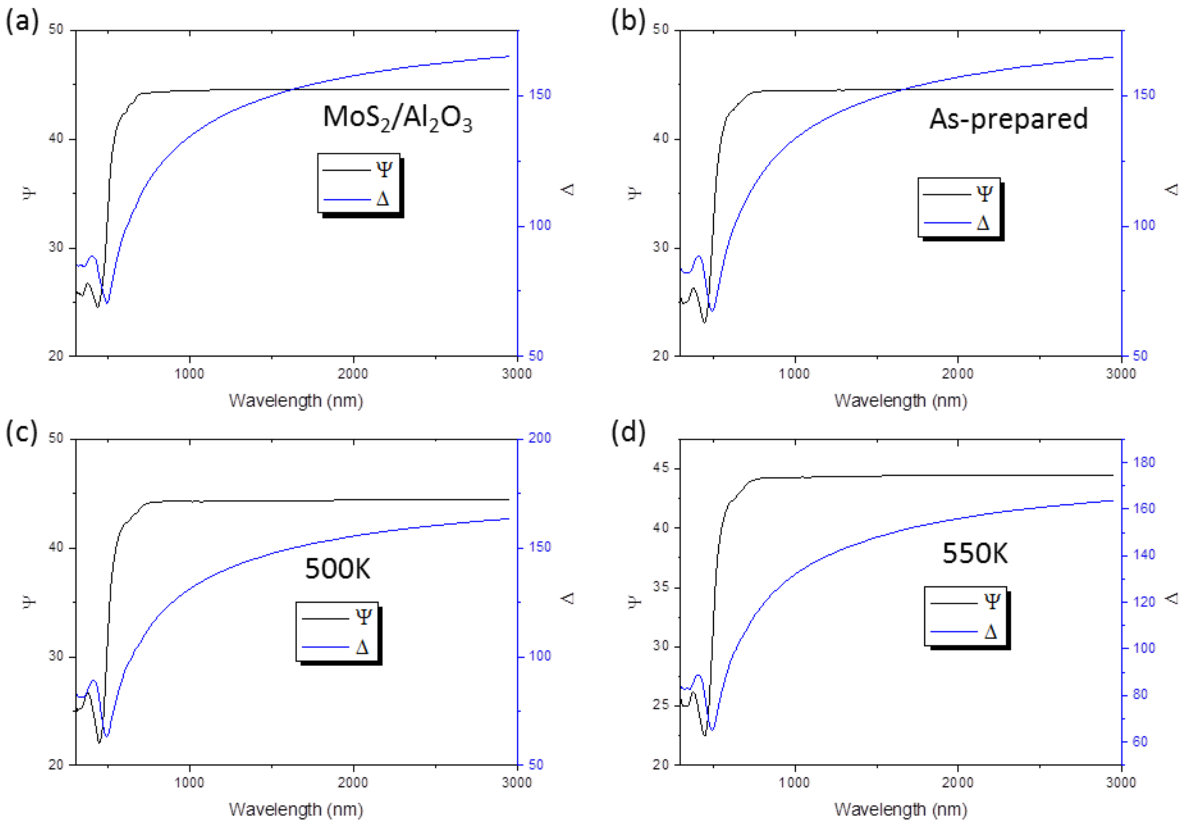

Figure S12. Ψ and Δ data of monolayer-MoS_2 in the near-IR-to-visible range. (a) MoS_2/Al_2O_3, (b) As-prepared MoS_2/Au, (c) MoS_2/Au after annealing at 500K and, (d) MoS_2/Au after annealing at 550K.

Supplementary Table

	MoS_2		WSe_2
1T' Fundamental Gap, E_g (µm)	11.5		14.4
Annealing Temperature (K)	500	550	500
Plasmon Position (µm)	16.2	17.8	12.7
ε_1 zero-crossing (µm)	16.3	18.1	12.8
Plasmon FWHM (µm)	1.78	3.33	2.68
Scattering Rate, $1/\tau$ (THz)	2.35	3.15	4.01
Dephasing Time, T (fs)	850	634	498

Table S1. Profiles of mid-infrared plasmons in 1T'-phase MoS_2 and WSe_2 monolayers.

Supplemental Methods:

Sample Preparation. High-quality large-area MoS_2 and WSe_2 monolayers are used in the high-resolution infrared-range spectroscopic ellipsometric experiments. The MoS_2 (WSe_2) atomic layers are synthesized on sapphire substrate (Al_2O_3) surface using the Chemical Vapour Deposition (CVD) technique with MoO_3 (WO_3) and S (Se) powders as reactants[1]. As for the gold substrate, a 200nm gold film is coated by sputtering technique on SiO_2/Si substrate. The CVD-grown MoS_2 (WSe_2) monolayer is then transferred onto the Au thin-film on SiO_2/Si substrate using polymethyl methacrylate (PMMA). Each sample is then annealed at a temperature of 80°C. This is to enhance the contact between the 2D-TMD film and the gold substrate and to eliminate any residues. The annealing

process of the respective monolayer sample is performed in a high vacuum chamber with a base pressure of 1×10^{-9}mbar at 500K and 550K, each for about 15mins. After annealing at the respective temperature, the sample is then naturally cooled to room temperature prior to each experimental measurement. Quality of the respective CVD-grown MoS_2 and WSe_2 atomic layers are confirmed using PL and Raman Spectroscopy.

High-resolution Transmission Electron Microscopy (HR-TEM) Characterization. To ensure that the samples are of good monolayer crystalline quality in its 1H-phase before annealing and 1T'-phase after annealing, HRTEM characterization is carried out for the monolayer-WSe_2/Au samples.

Figure S5 displays the HRTEM image of the as-prepared monolayer-WSe_2 (1H-phase) on Au before annealing. It shows that the monolayer sample is of premium quality where the W atoms are highly hexagonally ordered in its 1H-phase and the Se atoms in the upper layer are located directly above those on the lower layer. Therefore, there is only one Se atomic peak between two W atomic peaks (Figures S5b and S7b).

After annealing the sample at 500K, the distance between two W atoms (Figure S7b) is smaller (in the range between ~0.536(7) – 0.571(4) nm) than those before annealing (at ~0.580(5) nm). The trend of molecular distortion is also consistent with molecular structure as depicted in Figure S4, i.e., larger distance between adjacent W atoms for 1H/1T-phase (Figs. S4a and b, respectively) and shorter for 1T'-phase (Figs. S4c). Furthermore, the two Se atoms are located closer to one of the W atoms (Fig. S7c). This trend is consistent with the 1T' monolayer-WSe_2 structure (Figs. S4c and S7d) where the

W atoms are shifted horizontally in the direction indicated by the blue arrows. Conversely, in the ideal 1T-phase, the distances between two transition metal (W) atoms and that between the transition metal and the chalcogen atom (W-Se) should be the same (see ref. [2]).

Therefore, by comparing between the structural features of WSe_2 in its respective phases and the HR-TEM intensity profiles (Figures S4, S7c and d), we show that the W-atom distortions comparable to that of 1T'-phase in monolayer-WSe_2 are still present after annealing. The HR-TEM data supports our conclusion that structural phase transition has taken place with a partial distortion of monolayer-WSe_2 after annealing.

However, note that such characteristic 1T'-phase structural distortions are not sufficiently long-ranged for the signature zig-zag pattern of 1T'phase WSe_2 to be observed. Firstly, this is due to the coexistence of 1H and 1T' phases in our monolayer samples after the annealing process[3-4] which adds to the difficulty in imaging the zig-zag chains.

To further account for the difficulty in making clear observation of the characteristic zig-zag chains in our 1T'-phase monolayer-WSe_2, we clarify that it is difficult for HR-TEM characterization of the WSe_2 when it is on the Au substrate after the annealing process. This is because the electrons are strongly absorbed by the noble metal (Au) substrate.

Hence, HR-TEM imaging and characterization can only be performed by chemically transferring the monolayer-WSe_2 from the Au substrate after the annealing process to its free-standing state (N.B., this is not done for our optical spectroscopic and Raman, PL experiments). As a result of the lack of lattice strain exerted by the Au substrate, the 1T'

distortion of WSe_2 may be partially relaxed. This leads to difficulty in making the observation of the zig-zag patterns of the 1T'-phase WSe_2.

The main difference between the ideal 1T metallic phase and 1T' quasi-metallic phase is that the latter is inherently semiconducting with the presence of both the fundamental and inverted gaps that arise due to band inversion induced by spin-orbit coupling near the Fermi level (Figure S8)[5].

Hence, to distinguish our WSe_2 and MoS_2 monolayers, the optical data shows that for the 1T'-phase there are two gaps near the Fermi level and no obvious Drude response. This is an important set of evidence that our WSe_2/Au and MoS_2/Au samples are narrow-gap semiconducting after annealing. Moreover, the energies of the observed fundamental and inverted gaps (1T'-phase WSe_2: ~0.09 and ~0.74eV, respectively; 1T'-phase MoS_2: ~0.1 and ~0.49eV, respectively) are consistent with the calculated value of the respective 1T' phase. 2D-TMDs[5].

By combining the experimental results obtained from HR-TEM, optical spectroscopy, Raman, and PL techniques, we can conclude that there is indeed a 1H-1T' phase transition in our samples after the annealing process (see also Confirmation of 1H-1T' phase transition of WSe_2 and MoS_2 monolayers).

Confirmation of 1H-1T' phase transition of WSe_2 and MoS_2 monolayers. 1H-to-1T' phase transition of monolayer-WSe_2/Au is confirmed to have taken place by examining the evolution in the high-resolution photoluminescence and Raman, spectroscopy ellipsometric spectra (Figs. 2a, 2b and 2c, respectively in the main text) of WSe_2 brought

about by the annealing process. Initially, as observed in Fig. 2a in the main text, exciton peak A attributed to the direct band gap photoluminescence from the K-point at ~751nm is observed in the as-prepared sample[1]. By annealing the sample at high temperature, a similar phenomenon in the form of red-shift, broadening and intensity reduction of the peak take place which confirm the 1H-1T' phase transformation of monolayer-WSe_2[6]. This is further confirmed with the evolution in the Raman spectrum where the appearances of distinct peaks at positions such as ~136cm^{-1} and ~220cm^{-1} (labelled by arrows) not present in the as-prepared sample have been observed. Besides, main text Figure 2c displays the σ_1 spectrum where several strong and distinct absorption peaks related to various optical band transitions. Similar to the peaks observed via PL spectroscopy, peaks A (~750nm) and B (~600nm) are attributed to the direct gap transitions at the K/K'-points in the Brillouin zone of monolayer-WSe_2. These exciton peaks exhibit a slight redshift in the case of WSe_2/Au due to charge transfer from the gold substrate to the WSe_2 monolayer[7]. Besides, there is a broad near-infrared peak below exciton peak A at ~1675nm in WSe_2/Au. This optical feature is absent from WSe_2/Al_2O_3. At the annealing temperature of 550K, the intensity of the near-infrared peak is maximized. This is a further confirmation of the 1H-1T' phase transition of monolayer-WSe_2 where the energy position of the mid-infrared peak is close the theoretical value of the inverted gap of 1T'-WSe_2[5] as well as experimentally measured values of 1T'-phase monolayer-WSe_2 on Au and has been ascribed to the inverted gap [4].

The PL, Raman and ellipsometric data of monolayer-MoS_2/Au are displayed in main text Figs. 4a, 4b and 4c, respectively. The PL spectra indicate the presence of the prominent exciton peak A (~660nm) and the relatively weaker exciton peak B (~622nm). The

positions of these exciton peaks are in good agreement with previous reports on monolayer-MoS_2[8]. After annealing the sample at 500K and above, there is a drastic reduction in the intensity of both peaks A and B. This is an opposite effect from the previous PL study[9] and it also agrees with previous studies that involved the 1H-to-1T' structural phase transition of monolayer-MoS_2[3-4]. Figure 3b displays the Raman spectra of MoS_2/Au in its as-prepared state and upon annealing at 500 and 550K. In the two latter Raman spectra, the three main characteristic Raman active features at ~152 (J1), ~221 (J2) and ~314 cm^{-1} (J3) which are characteristic features of the 1T'-phase octahedral structure[10] can be observed. The consistency between the Raman features with previous theoretical and experimental studies confirms the 1H-1T' phase transition of monolayer-MoS_2[11]. Similar to the WSe_2/Au system after annealing, the σ_1 spectra (main text Figure 4c) as measured by spectroscopic ellipsometry exhibit a broad near-infrared peak below the excitonic peak A with its intensity maximized at the annealing temperature of 500K. This is absent from MoS_2/Al_2O_3. Likewise, the energy position of the near-infrared peak at ~2500 nm (indicated by arrow) is again similar to the theoretically determined value of the inverted gap of 1T'-phase monolayer-MoS_2[5] as well as previously experimentally determined values[3-4]. Therefore, the collective detection of this inverted gap via the means of high-resolution PL, Raman and spectroscopic ellipsometry along with the observation of the fundamental gap (main text Fig. 4c) confirms the presence of the 1T'-phase monolayer-MoS_2 after the high-temperature annealing process.

High-resolution Infrared-range Spectroscopic Ellipsometry. High-resolution optical spectra of MoS_2 and WSe_2 monolayers are measured using a J.A Woollam Co., Inc. IR-

VASE spectroscopic ellipsometer in the spectral region of the respective 2D-TMD fundamental gap with at a wavelength range of 8—18.2 μm for monolayer-MoS_2/Au and 6.8-15.5 μm monolayer-WSe_2/Au. The near-infrared to visible range optical data are separately measured using Woollam VASE Ellipsometer with a wavelength range of ~300-3000nm.Ellipsometric parameters Ψ (ratio between the amplitude of *p*- and *s*-polarized reflected light) and Δ (phase difference between of *p*- and *s*-polarized reflected light) are measured. Measurements by the IR-VASE and the VASE Ellipsometer have a spectral resolution of ~15cm^{-1} (~2 meV) with the incident beam having a spot size of ~0.4mm×~1mm. The optical conductivity, σ_1, dielectric coefficient, ε_1, and dynamic loss-function, of the respective monolayers are extracted from the parameters Ψ and Δ using an air/MoS_2/Au (or air/WSe_2/Au) multilayer model [4], where the monolayer-MoS_2 (WSe_2) comprises an average homogeneous and uniform medium.

Ellipsometric parameters are defined as

$$\tan\Psi \exp(i\Delta) \equiv \frac{r_p}{r_s} \qquad (S1)$$

where $r_{p(s)}$ denotes the reflectivity of the *p*-(*s*-) polarized light. Using Fresnel equations, the variables are defined

$$r_p^{ij} = \frac{n_j \cos\theta_i - n_i \cos\theta_j}{n_j \cos\theta_i + n_i \cos\theta_j} \qquad \text{(S2)}$$

and

$$r_s^{ij} = \frac{n_j \cos\theta_i - n_i \cos\theta_j}{n_j \cos\theta_i + n_i \cos\theta_j} \qquad \text{(S3)}$$

n and θ represent the complex refractive index and the angle of incident, respectively. The i and j represent the two materials in each layer. The dielectric function $\varepsilon(\omega) = \varepsilon_1(\omega) + i\varepsilon_2(\omega)$ of the material are derived using

$$\sqrt{\varepsilon(\omega)} = n(\omega) \qquad (S4)$$

where ω denotes the photon frequency.

The reflectivity of monolayer-MoS_2 (WSe_2) film on substrate is expressed as ,

$$r_{multi} = \frac{r_{amb,WS_{e2}} + r_{WS_{e2},sub} \exp(i2\delta_{WS_{e2}})}{1 + r_{amb,WS_{e2}} \cdot r_{WS_{e2},sub} \exp(i2\delta_{WS_{e2}})} \qquad (S5)$$

where

$$\delta_{WS_{e2}} = \frac{2\pi d_{WS_{e2}}}{\lambda}\sqrt{n_{WS_{e2}}^2 - n_{amb}^2 \sin^2\theta} \qquad (S6)$$

Subscripts *multi* and *amb* represent the WSe_2 on Au substrate multilayer system and the ambient, respectively, while δ_{WSe2} is the change in light phase as it reflects off the WSe_2 film, and d_{WSe2} is the thickness of the WSe_2 film. d_{MoS2} (d_{WSe2}) is used 0.7nm for the average of thickness of MoS_2 (WSe_2) monolayer grown by CVD. For the refractive index of the substrate, the spectroscopic ellipsometry were measured separately. ellisometric measurements are performed at 70-degree incident angle for temperature-dependent measurements. The dielectric coefficient of MoS_2 (WSe_2) monolayer is obtained through direct function inversion of Equation S4 after fitting.

The original Ψ and Δ data for both WSe_2/Au and MoS_2/Au samples in their pristine state and after the annealing processes have been included in Figures S9 to S12.

Analysis of anistropic plasmon in 1T'-phase 2D-TMDs. Frequency-dependent dielectric functions are been derived based on the Drude Model:

$$\varepsilon(\omega) = \varepsilon_1(\omega) + i\varepsilon_2(\omega) = 1 - \frac{\omega_p^2}{\omega^2 - i\omega/\tau} \qquad \text{(S7)}$$

The scattering rate, 1/τ, of plasmons are estimated using the energy differences between the plasmon energy, ω_p, from the loss function spectrum, and the ε_1 zero-crossing, $\omega_{\varepsilon 1=0}$, using the following [12]

$$\frac{1}{\tau} = \sqrt{\omega_p^2 - \omega_{\varepsilon_1=0}^2} \qquad \text{(S8)}$$

Plasmon dephasing time, T, is then estimated based on the following:

$$T = 2\tau \qquad \text{(S9)}$$

To evaluate the possible functionality of anisotropic plasmonics in 1T'-phase 2D-TMDs, we performed further analysis based on the LF spectra of both 1T'-phase MoS_2 and WSe_2 monolayers. Fitting analyses show that the MoS_2/Au plasmon has a width of ~1.77 μm while that of WSe_2/Au is broader at ~2.68 μm (see Table S1 for details). The peak widths are similar to plasmons reported in noble metals [13]. These widths are also similar in magnitude as mid-infrared plasmons reported in graphene nanostructures [14] and metallic TMD system of 2H-$TaSe_2$ [15].

As compiled in Table S1, note that a slight discrepancy is observed between the LF peak position and the ε_1 zero-crossing and it can be attributed to the presence of charge scattering resulted by the interaction between the plasmon modes and the 2D-TMD lattice.

Based on this disparity, estimated free-electron scattering rate, $1/\tau$, in monolayer-MoS_2 is at ~2.35 THz after annealing at 500K and ~3.15THz after annealing at 550K. Free-electron scattering in 1T'-phase WSe_2 after annealing at 550K is also postulated for monolayer-MoS_2 at ~4.01 THz. The plasmon dephasing times, T, of each system are further derived to be ~850 fs (500K) and ~634 fs (550K) for MoS_2 and ~498 fs for WSe_2 after annealing at 550K.

While the widths of the plasmons in both MoS_2 and WSe_2 monolayers have similar magnitudes compared to other systems, the dephasing times, T, of these plasmons are significantly higher than those found in other systems. Theoretical studies have predicted that dephasing times for plasmons in noble metals are significantly smaller at ~5-12 fs [15]. Experimental measurements of nano-metallic systems such as gold nanorods have plasmon dephasing times up to ~31 fs [16]. In the case of 2D graphene nanostructures, plasmon lifetimes have been observed to be in the magnitude of 20 fs and below [14]. The dephasing times of plasmons found in these systems are significantly smaller than those reported in 1T'-phase MoS_2 and WSe_2 monolayers. Unlike conventional metal plasmons whose application range is mainly in the visible regime, the operation of anisotropic plasmon in 1T'-phase in the mid-infrared regime promises novel photonic applications in disciplines relating to nano-plasmonics such as topological protection[17], nano-lasing technologies[18], atomic-scale dipole-forbidden absorption[19] and other heterostructure engineering applications in the optical and near-infrared regime. More importantly, with the significantly longer plasmon lifetimes, 1T'-phase TMD monolayers have greater potential for novel device applications. Insights pertaining the plasmonic dephasing time

and lattice interaction are pivotal to the understanding of possible mitigating factors to reduce plasmonic dissipation and losses resulted through the interaction between the plasmon modes and the 2D-TMD lattice.

Comparison of plasmon position with other systems. We compare this anisotropic plasmon in both 1T'-phase MoS_2 and WSe_2 monolayers in the mid-infrared regime with those found in other systems. In other TMD systems such as layered 1H-phase $TaSe_2$, $NbSe_2$, and TaS_2, the plasmon frequencies are significantly higher and they fall within the near infrared and visible electromagnetic regime (~1eV) [15]. Plasmon frequencies detected in noble metals are found in the violet and near-ultraviolet electromagnetic region (~3.7eV) [13]. Plasmons in graphene nanostructures are detected in the mid-infrared regime at ~4—15 μm [14]. While this spectral region offers important device functionalities ranging from biological sensors to surface-enhanced spectroscopies, graphene's vanishingly low on/off ratio due to its zero bandgap renders it ineffective in multiple device applications. Therefore, 2D-TMDs emerge as a superior alternative in the fabrication of plasmonic devices. Particularly, with growing interest in plasmonic properties in the infrared regime, the observation of plasmon in 1T'-phase monolayer-WSe_2 in this electromagnetic region is an immensely important breakthrough where crucial fingerprints of molecular vibrational modes are found and where plasmonics can be exploited in multiple scientific and engineering applications [20].

Plasmonic Mediation with Superconductivity of 2D-TMDs. In a recent study, inter-planar Coulomb interaction is identified to result in the coherent out-of-plane (*z*-direction) acoustic plasmon in copper oxide (cuprates) based superconductors [21]. This out-of-plane coupling between the adjacent CuO_2-planes and the propagation of the *z*-directional plasmons is suggested to play a crucial role in mediating superconductivity in multilayer cuprate superconductors. With the interlayer dynamic charge coupling taking place between two-dimensional CuO_2-planes in three-dimensional cuprate lattices, such an analogous phenomenon involving reduced dimensionality is also noticeable here where coupling between one-dimensional zig-zag chains in 2D-TMD systems occurs. Hence, the coupling between the adjacent zig-zag transition metal chains in 1T'-phase monolayer-WSe_2 drives the collective charge dynamics along the *y*-direction (Fig. 1a) eventually results in the anisotropic plasmon. The notion that long-range inter-chain coupling leads to anistropic plasmon formation is further substantiated by reports that long-range electronic correlations results in the appearance of plasmons in other strongly-correlated systems [22].

This study holds implications in unravelling the mechanism that governs superconductivity. Multiple models have been proposed to account for the formation of electron pairs. This includes the resonant valence bond [23], electron-phonon interaction[24], and the spin fluctuation [25] theories that have been proposed to mediate high-temperature superconductivity. While charge-lattice coupling (electron-phonon interactions) could play a critical role in overcoming the strong electronic repulsion and leads to the formation of Cooper pairs in conventional superconductivity [26], electron-electron

coupling in the form of spin fluctuations [25] may also play a part in facilitating the onset of unconventional superconductivity via the formation of electron pairs. Similarly, with suggestions of inter-planar acoustic plasmons playing a substantial role in mediating high-temperature superconductivity [27], we postulate that the anisotropic plasmon observed to propagate perpendicular to the zig-zag transition metal atomic chains in monolayer-WSe_2 should serve as an electron-plasmon coupling mechanism in the formation of electron pairings that possibly underlies the superconductive phenomenon in 1T'-phase 2D-TMDs [28].

First-principles Calculations. All density-functional theory (DFT) based calculations are conducted out using Vienna ab initio simulation package (VASP 5.4.4.18)[29]. The Perdew-Burke-Ernzerhof (PBE) format exchange-correlation functionals and the projector augmented wave (PAW) potentials have been used[30]. The cutoff energy for the electronic plane-wave expansion has been set to 500eV. A 20Å vacuum layer has also been applied perpendicular to 1T'-phase monolayer-WSe_2 surface. 9×12×1 *k*-point meshes have been used to sample the Brillouin zone. The energy and force on each atom were converged at values below 1.0×1.0^{-8} eV and 0.01eV/Å, respectively. SOC effects have also been included in the calculations. Based on these settings and parameters, the calculated lattice constants of 1T'-phase monolayer-WSe_2 are a=5.96 and b=3.31 Å – in good agreement with previous studies[31]. The dielectric matrices were calculated using random phase approximation (RPA) on the top of the G_0W_0. Of which, 216 empty bands have been included. The energy cutoff for the response function has been set to be 220

eV, and 40 highest valence bands and 6 lowest conduction bands were used as the basis for the excitation eigenstates. The energy LF is calculated based on the expression

$$Im[-1/\varepsilon_M]$$

Supplementary References

[1] J.-K. Huang, J. Pu, C.-L. Hsu, M.-H. Chiu, Z.-Y. Juang, Y.-H. Chang, W.-H. Chang, Y. Iwasa, T. Takenobu, L.-J. Li, *ACS Nano* **2014**, *8*, 923.

[2] G. Eda, T. Fujita, H. Yamaguchi, D. Voiry, M. Chen, M. Chhowalla, *ACS Nano* **2012**, *6*, 7311.

[3] X. Yin, Q. Wang, L. Cao, C. S. Tang, X. Luo, Y. Zheng, L. M. Wong, S. J. Wang, S. Y. Quek, W. Zhang, A. Rusydi, A. T. S. Wee, *Nat. Commun.* **2017**, *8*, 486.

[4] X. Yin, C. S. Tang, D. Wu, W. Kong, C. Li, Q. Wang, L. Cao, M. Yang, Y.-H. Chang, D. Qi, F. Ouyang, S. J. Pennycook, Y. P. Feng, M. B. H. Breese, S. J. Wang, W. Zhang, A. Rusydi, A. T. S. Wee, *Adv. Sci.* **2019**, *6*, 1802093.

[5] X. Qian, J. Liu, L. Fu, J. Li, *Science* **2014**, *346*, 1344.

[6] D. Voiry, A. Mohite, M. Chhowalla, *Chem. Soc. Rev.* **2015**, *44*, 2702.

[7] K. F. Mak, K. He, C. Lee, G. H. Lee, J. Hone, T. F. Heinz, J. Shan, *Nat. Mater.* **2012**, *12*, 207.

[8] K. F. Mak, C. Lee, J. Hone, J. Shan, T. F. Heinz, *Phys. Rev. Lett.* **2010**, *105*, 136805.

[9] G. Eda, H. Yamaguchi, D. Voiry, T. Fujita, M. Chen, M. Chhowalla, *Nano Lett.* **2011**, *11*, 5111.

[10] Y. Kang, S. Najmaei, Z. Liu, Y. Bao, Y. Wang, X. Zhu, N. J. Halas, P. Nordlander, P. M. Ajayan, J. Lou, Z. Fang, *Adv. Mater.* **2014**, *26*, 6467.

[11] A. P. Nayak, T. Pandey, D. Voiry, J. Liu, S. T. Moran, A. Sharma, C. Tan, C.-H. Chen, L.-J. Li, M. Chhowalla, J.-F. Lin, A. K. Singh, D. Akinwande, *Nano Lett.* **2015**, *15*, 346.

[12] S. A. Maier, *Plasmonics: fundamentals and applications*, Springer Science & Business Media, **2007**.

[13] M. Rocca, F. Moresco, U. Valbusa, *Phys. Rev. B* **1992**, *45*, 1399.

[14] H. Yan, T. Low, W. Zhu, Y. Wu, M. Freitag, X. Li, F. Guinea, P. Avouris, F. Xia, *Nat. Photonics* **2013**, *7*, 394.

[15] J. van Wezel, R. Schuster, A. König, M. Knupfer, J. van den Brink, H. Berger, B. Büchner, *Phys. Rev. Lett.* **2011**, *107*, 176404.

[16] T. Zhao, J. W. Jarrett, J. S. Johnson, K. Park, R. A. Vaia, K. L. Knappenberger, *J. Phys. Chem. C* **2016**, *120*, 4071.

[17] D. Jin, T. Christensen, M. Soljačić, N. X. Fang, L. Lu, X. Zhang, *Phys. Rev. Lett.* **2017**, *118*, 245301.

[18] Y.-J. Lu, J. Kim, H.-Y. Chen, C. Wu, N. Dabidian, C. E. Sanders, C.-Y. Wang, M.-Y. Lu, B.-H. Li, X. Qiu, W.-H. Chang, L.-J. Chen, G. Shvets, C.-K. Shih, S. Gwo, *Science* **2012**, *337*, 450.

[19] N. Rivera, I. Kaminer, B. Zhen, J. D. Joannopoulos, M. Soljačić, *Science* **2016**, *353*, 263.

[20] Z. Fei, A. S. Rodin, G. O. Andreev, W. Bao, A. S. McLeod, M. Wagner, L. M. Zhang, Z. Zhao, M. Thiemens, G. Dominguez, M. M. Fogler, A. H. C. Neto, C. N. Lau, F. Keilmann, D. N. Basov, *Nature* **2012**, *487*, 82.

[21] M. Hepting, L. Chaix, E. W. Huang, R. Fumagalli, Y. Y. Peng, B. Moritz, K. Kummer, N. B. Brookes, W. C. Lee, M. Hashimoto, T. Sarkar, J. F. He, C. R. Rotundu, Y. S. Lee, R. L. Greene, L. Braicovich, G. Ghiringhelli, Z. X. Shen, T. P. Devereaux, W. S. Lee, *Nature* **2018**, *563*, 374.

[22] E. G. C. P. van Loon, H. Hafermann, A. I. Lichtenstein, A. N. Rubtsov, M. I. Katsnelson, *Phys. Rev. Lett.* **2014**, *113*, 246407.

[23] P. W. Anderson, *Science* **1987**, *235*, 1196.

[24] J. Lee, K. Fujita, K. McElroy, J. A. Slezak, M. Wang, Y. Aiura, H. Bando, M. Ishikado, T. Masui, J. X. Zhu, A. V. Balatsky, H. Eisaki, S. Uchida, J. C. Davis, *Nature* **2006**, *442*, 546.

[25] D. J. Scalapino, *Rev. Mod. Phys.* **2012**, *84*, 1383.

[26] J. Bardeen, L. N. Cooper, J. R. Schrieffer, *Phys. Rev.* **1957**, *108*, 1175.

[27] V. Z. Kresin, H. Morawitz, *Phys. Rev. B* **1988**, *37*, 7854.

[28] Y. Qi, P. G. Naumov, M. N. Ali, C. R. Rajamathi, W. Schnelle, O. Barkalov, M. Hanfland, S.-C. Wu, C. Shekhar, Y. Sun, V. Süß, M. Schmidt, U. Schwarz, E. Pippel, P. Werner, R. Hillebrand, T. Förster, E. Kampert, S. Parkin, R. J. Cava, C. Felser, B. Yan, S. A. Medvedev, *Nat. Commun.* **2016**, *7*, 11038.

[29] G. Kresse, J. Hafner, *Phys. Rev. B* **1993**, *47*, 558.

[30] P. E. Blöchl, *Phys. Rev. B* **1994**, *50*, 17953.

[31] K.-A. N. Duerloo, Y. Li, E. J. Reed, *Nat. Commun.* **2014**, *5*, 4214.
